# Depot‐Specific Insulin Receptor Isoform Dynamics During Human Adipose‐Derived Stem Cell Adipogenesis

**DOI:** 10.1002/oby.70315

**Published:** 2026-10-01

**Authors:** Valentina Annamaria Genchi, Celeste Lauriola, Cristina Caccioppoli, Rossella D'Oria, Nicola Marrano, Giuseppina Biondi, Sebastio Perrini, Annalisa Natalicchio, Luigi Laviola, Francesco Giorgino, Angelo Cignarelli

**Affiliations:** ^1^ Department of Precision and Regenerative Medicine and Ionian Area‐Section of Internal Medicine, Endocrinology, Andrology and Metabolic Diseases University of Bari Aldo Moro Bari Italy; ^2^ Section of Endocrinology, Department of Medicine and Surgery LUM University Casamassima Bari Italy

**Keywords:** adipogenesis, human adipose stem cells, insulin receptor isoform

## Abstract

**Objective:**

The insulin receptor (IR) and the insulin‐like growth factor I receptor (IGF‐IR) regulate adipose tissue (AT) differentiation. This study investigated the expression of IGF‐IR, IR, and the IR isoforms (IRa and IRb) in human subcutaneous (S‐ASC) and visceral (V‐ASC) adipose‐derived stem cells.

**Methods:**

S‐AT and V‐AT biopsies were obtained from human donors and ASC were differentiated into adipocytes. mRNA levels of all receptors were quantified by qPCR at baseline and after differentiation. IR protein expression and adipogenic efficiency were assessed by flow cytometry.

**Results:**

In undifferentiated ASC, IGF‐IR transcripts exceeded IR and were higher in S‐ASC than V‐ASC. Upon differentiation, IGF‐IR decreased in S‐ASC but remained unchanged in V‐ASC, whereas IR increased in both depots. S‐ASC displayed ~twofold higher IRa expression and greater insulin‐induced Akt activation. During adipogenesis, IR expression rose in both depots, with IRb exceeding IRa particularly in V‐ASC (+50%). In whole AT, IR exceeded IGF‐IR, and no depot‐specific differences were observed between IR isoforms. Adipogenic conversion was higher in S‐ASC (60%) than V‐ASC (20%). Only ~77% of S‐ASC‐ and ~61% of V‐ASC‐derived adipocytes expressed surface IR.

**Conclusions:**

Depot‐specific dynamics of IR isoforms may influence adipogenic capacity and insulin responsiveness and suggest partially receptor‐independent pathways of adipocyte differentiation.

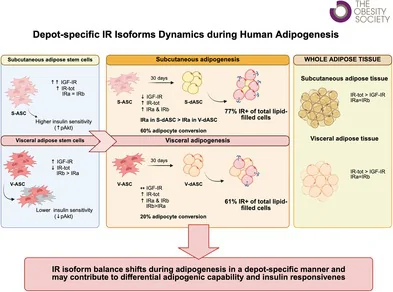

## Study Importance

- What is already known?
  ○ Insulin receptor (IR) signaling is required for adipocyte differentiation, whose activation displays intrinsic functional differences in subcutaneous and visceral adipose depots.
  ○ IR consists in two splice isoforms (IRa and IRb) with different signaling properties, but their quantitative distribution in human adipose‐derived stem cells from both depots is still unexplored.
- What does this study add?
  ○ The study provides an absolute quantification of IGF‐IR, total IR, and IR isoforms mRNA in human subcutaneous and visceral adipose stem cells, identifying depot‐specific receptor signatures and adipogenesis‐associated isoform shifts.
  ○ Cellular heterogeneity in mature adipocytes was observed, including IR‐negative lipid‐filled cells, suggesting alternative pathways supporting adipogenesis.
- How might these results change the direction of research?
  ○ These findings identify IR isoform balance as a previously underexplored layer of regulation in human adipogenesis.
  ○ The presence of IR‐independent adipocyte differentiation challenges current receptor‐centric models and supports investigation of parallel signaling networks controlling adipose development.

## Graphical abstract

Absolute quantification reveals depot‐specific insulin receptor (IR) isoform signatures in human adipose stem cells, with subcutaneous cells enriched in IRa and exhibiting greater adipogenic capacity and insulin responsiveness. The emergence of IR‐negative adipocytes highlights partially receptor‐independent pathways regulating human adipogenesis.

## 1 Introduction

Insulin is a potent adipogenic hormone inducing a series of transcription factors involved in differentiation of preadipocytes into mature adipocytes. The actions of insulin on adipose tissue (AT) are principally mediated by the insulin receptor (IR) whose extent of activation varies according to fat anatomical depot. Indeed, visceral AT (V‐AT) typically shows higher rates of insulin‐induced glucose uptake as compared to subcutaneous AT (S‐AT) regardless of BMI [1, 2].

In a mice model, genetic abrogation of IR was associated with severe lipodystrophy in association with impaired suppression of lipolysis, altered glucose levels [3, 4], and other metabolic abnormalities [5], all conditions leading to a decreased life‐span, further illustrating the key role of insulin in this tissue. Insulin activates key mechanisms regulating AT expansion through adipogenesis and lipogenesis of the resident adipocytes [6].

The adipose‐derived stem cells (ASC) represent the multipotent staminal component of AT that contributes to regulation of AT expansion and cellular turnover [2, 6, 7] and whose adipogenic features change according to genetic background, diet, and fat depot [2, 7, 8]. Recent evidence showed that both IR and insulin‐like growth factor I receptor (IGF‐IR) are essential for regulating AT functions; in mice, genetic disruption of both IR and IGF‐IR in AT leads to decreased AT mass and failure of adipocyte differentiation and counteracts diet‐induced metabolic abnormalities [5].

Notably, the effects of IR activation on AT may also depend on the relative expression of its isoforms. IR consists of two isoforms which differ based on the inclusion or exclusion of 12 amino acids in the C‐terminal domain [9]. IRa, generated by the skipping of exon 11, is mostly expressed in undifferentiated cells and promotes prenatal development and tissue growth [10], whereas IRb, including exon 11, exerts preferentially metabolic actions especially in insulin‐sensitive tissues [11, 12, 13]. Considering the specificity of IR splice variants in activating mitogenic and metabolic arms of insulin signaling, their differential expression may affect AT physiology and functions. However, few studies investigated the expression of IR isoforms in human AT, and the expression of these variants in subcutaneous ASC (S‐ASC) and visceral ASC (V‐ASC) in relation to their differentiation into mature adipocytes is unknown.

## 2 Methods

### 2.1 Reagents

Human insulin was purchased from Roche Diagnostics. Rosiglitazone was kindly provided by GlaxoSmithKline. Anti‐phospho‐Akt Ser473 (pAkt^S473^; cat. #4060S), anti‐phospho‐Akt Thr308 (pAkt^T308^; cat. #9275L), anti‐total Akt (cat. #9272), anti‐phospho‐Erk T202/Y204 (pErk^T202/Y204^; cat. #9101S), and anti‐total Erk (cat. #9102) were all from Cell Signaling Technology. Anti‐CD220‐APC was purchased from Miltenyi Biotec and used for the detection of IR protein expression by flow cytometry assay. All tissue culture reagents were from Life Technologies. Reagents for SDS‐PAGE were supplied by Bio‐Rad. All other chemicals were purchased from Sigma Chemical Co. unless otherwise stated.

### 2.2 ASC Isolation and Culture

Unpaired biopsies of abdominal subcutaneous adipose tissue (S‐AT), obtained from the periumbilical region, and visceral adipose tissue (V‐AT), obtained from the greater omentum, were collected from 54 human participants during laparoscopic bariatric surgery (sleeve gastrectomy) or laparoscopic cholecystectomy, as previously described [8]. The sampling site was consistent among all participants. The cohort (*N* = 54; 36 females and 18 males) had a mean BMI of 35.0 ± 7.9 kg/m^2^ and a mean waist circumference of 109.6 ± 18.5 cm, indicating a large prevalence of central obesity (77%). Mean HbA1c was 54.8 ± 31.7 mmol/mol (≈7.17%); mean fasting plasma glucose was 113 ± 39.2 mg/dL and mean HOMA‐IR was 5.9 ± 5.6, consistent with a high prevalence of insulin resistance and type 2 diabetes in this population (~40%) (Table S1). All participants gave their informed consent for inclusion before they participated in the study. The study was approved by the Ethics Committee at the University of Bari (approval no. 152/2012). ASC were isolated and differentiated into adipocytes as previously described [8, 14, 15, 16].

### 2.3 Oil‐Red‐O Staining of Triglyceride‐Containing Cells

Oil‐Red‐O staining of differentiated adipocytes was performed to monitor and obtain a quantitative measure of lipogenesis, as previously described [7, 8, 14]. The stained lipid droplets were visualized by light microscopy. The measurement of accumulated triglycerides was obtained by evaluating the optical density at 510 nm of the eluted Oil‐Red‐O stain using an iMark Microplate Reader (Bio‐Rad).

### 2.4 Nile Red Staining and Assessment of ASC Conversion to Adipocytes

Fixed cells were stained with Nile Red according to a published protocol [7, 8, 17] using DAPI as nuclear counterstain. The adipocyte conversion rate was assessed by counting the cells that had accumulated lipid droplets with the imaging software package CellProfiler (available from Broad Institute at www.cellprofiler.org). The CellProfiler software requires fluorescence spots on a dark background, and this makes it necessary to use Nile Red instead of Oil‐Red‐O as stain for lipids. Data represent the proportion of positive versus total cells (mean of 10 random microscopic fields).

### 2.5 Total RNA Extraction, Reverse Transcription, and Absolute Quantitative PCR

Total RNA was extracted from whole AT using the phenol/chloroform method and from ASC and differentiated adipocytes by cell lysis supplemented with β‐mercaptoethanol, followed by purification with a RNeasy column (QIAGEN). A total of 500 ng of RNA was reverse transcribed using random hexamers and reverse transcription (RT) reagents from Applied Biosystems. Specific primers (Table S2) were designed using Primer Express 3.0 (Applied Biosystems) and synthesized by Eurofins Genomics. Absolute quantitative PCR (qPCR) was performed by using gene block standards for IGF‐IR (95 bp), IR‐tot (99 bp), IRa (114 bp), and IRb (114 bp) purchased by Eurofins Genomics (Table S3) and reconstituted in nuclease‐free water. The concentrations of each gene block determined by NanoDrop spectrophotometry were as follows: IGF‐IR 76.4 ng/μL, total IR (IR‐tot) 79.9 ng/μL, IRa 9.95 ng/μL, and IRb 5.30 ng/μL. The corresponding copy numbers were calculated assuming 660 g/mol per base pair and using Avogadro's number (6.022 × 10^23^ mol^−1^) (Table S3) [18]. For each target, a nine‐point standard curve (S1–S9) spanning 1 × 10^9^ to 1 × 10^1^ copies/μL was generated by serial 1:10 dilutions. qPCR reactions were performed by using SsoAdvanced Universal SYBR Green Supermix (Bio‐Rad). Data were analyzed using CFX Maestro software (v 2.3, Bio‐Rad) in “Absolute Quantification” mode. Sample concentrations (copies/μL) were interpolated from the standard curves (Ct vs. log_10_[copies/μL]). Curve efficiencies ranged from 92% to 105% with *R* ^2^ > 0.99 for all targets, consistent with MIQE guidelines [18].

### 2.6 Analysis of Insulin Signaling

ASC at baseline and after acute insulin stimulation were lysed in buffer supplemented with protease and phosphatase inhibitor mixtures (Roche Applied Science). SDS‐PAGE and immunoblotting were carried out as previously described [7, 8, 19]. Protein bands were quantified using QuantityOne image analysis software (Bio‐Rad).

### 2.7 Quantification of IR Protein Expression During Adipogenesis by Flow Citometry Assay

To assess IR protein expression according to stage of adipogenesis, ASC from both depots were harvested before (Day 0) and after adipocyte differentiation (Day 16 and 30) by using a blunted cell dissociation reagent (StemPro accutase, Thermo Fisher). Then the cells were washed in sterile PBS 1X and pelleted at 1100 rpm × 5 min. The pellets were resuspended in PBS 1X to determine cell number by using Automated Cell Counter (Bio‐Rad). After centrifugation at 1100 rpm × 5 min, the cells were resuspended in antibody solution (PBS 1X, 0.5% bovine serum albumin [BSA], and 2mM EDTA) containing anti‐CD220‐APC (1:10) and incubated at +4°C in the dark for 10 min. Next, the cells were centrifugated at 1100 rpm × 5 min and stained by Nile Red solution (20 ng/mL in PBS 1X) at +4°C for 15 min, as described in a previous study [20]. After final centrifugation at 1100 rpm x 5 min, the cells were resuspended in 500 μL of PBS 1X and analyzed by Flow Cytometer Navios (Beckman Coulter). Anti‐CD220‐APC was excited at 594/633 nm, and fluorescence emission was collected with the FL6 detector (660 nm). Nile Red was excited with the 488 nm laser, and emission was detected with the FL2 channel (575 nm). For all conditions (Day 0–30), unstained cells were used as negative controls to set fluorescence thresholds. Flow cytometry data were analyzed using Kaluza software (Beckman Coulter). Detection sensitivity for IR was determined using unstained negative control ASC acquired under the same instrument settings and gating strategy as stained samples (30,000 events per sample). The limit of blank (LoB) was calculated as the mean plus 1.645 × standard deviation (SD) of IR^+^ events in negative controls, and the limit of detection (LoD) was estimated as LoB plus one × SD. The LoD corresponded to ~0.7% IR^+^ cells (≈210 events), reflecting background autofluorescence and gating. Values below this threshold were considered indistinguishable from background. Only gated, viable, single cells were analyzed. Gates for Nile Red^+^ cells were defined based on the fluorescence distribution of unstained controls, and the percentage of Nile Red^+^ cells was calculated within the gated, viable, single‐cell population for each experimental condition [21].

### 2.8 Statistical Analysis

Experimental results are shown as mean ± standard error of the mean (SEM). Normality of distribution was evaluated by Shapiro–Wilk test to choose appropriate statistical analyses.

Student's *t*‐test and Mann–Whitney test were used for comparing two groups with normal and non‐normal distributed data, respectively. The analysis of multiple comparison was performed by Kruskal–Wallis test followed by Dunn's multiple comparisons test for non‐normally distributed data. For evaluation of two independent variables, two‐way ANOVA followed by Sidak's multiple comparisons test was applied.

Multiple Mann–Whitney tests with Holm‐Sidak correction were applied for analysis of differences between S‐ASC and V‐ASC across multiple insulin concentrations. Log‐transformation of dataset was applied after verifying normalized distribution of data by Shapiro–Wilk test. Dose–response curves were fitted by nonlinear regression using a four‐parameter logistic model (variable slope, bottom = 0 and top = 100 constrained), and EC_50_ values were calculated for each cell type. Comparison of EC_50_ values between S‐ASC and V‐ASC was performed by *F*‐test on the LogEC_50_ parameter. Statistical significance was set at *p* < 0.05 for all analyses. All statistical analyses were performed using GraphPad Prism 11 and RStudio Workbench (version 2022.02.2).

## 3 Results

### 3.1 Differential Expression of IGF‐IR and IR Isoforms in S‐ASC and V‐ASC

Absolute qPCR with standard curves was performed to quantify IGF‐IR, IR‐tot, and the splice variants IRa and IRb in ASC derived from human subcutaneous and visceral fat.

In both depots, IGF‐IR was the most abundant transcript, significantly exceeding IR‐tot levels (Figure 1a,b). In S‐ASC, IGF‐IR copy number was significantly higher than IR‐tot (~1.5 × 10^4^ ± 0.20 × 10^4^ vs. ~4.2 × 10^3^ ± 0.54 × 10^3^ copies/μL; *p* < 0.05; Figure 1a). Similarly, in V‐ASC, IGF‐IR was significantly more abundant than IR‐tot (~1.0 × 10^4^ ± 0.13 × 10^4^ vs. ~2.7 × 10^3^ ± 0.37 × 10^3^ copies/μL; *p* < 0.01; Figure 1b), whereas IRb also significantly exceeded IRa in V‐ASC (IRb: 2.0 × 10^3^ ± 0.18 × 10^3^ vs. IRa: 1.3 × 10^3^ ± 0.15 × 10^3^ copies/μL; *p* < 0.05; Figure 1b).

**FIGURE 1:**
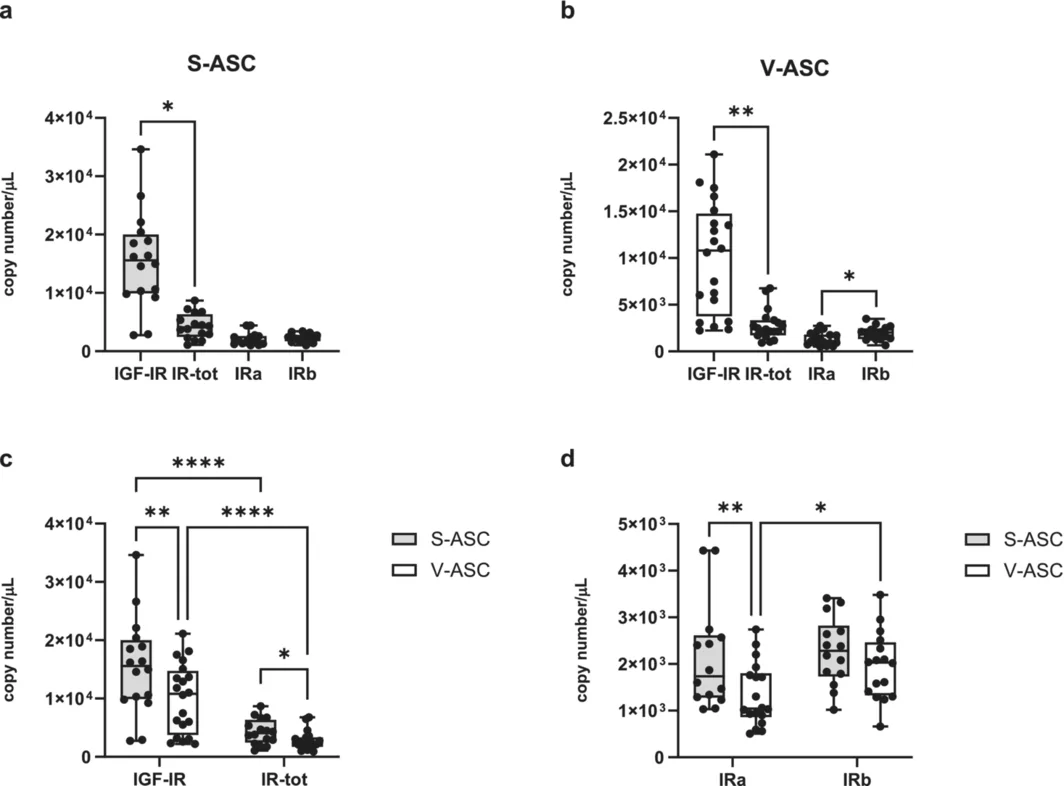
Quantification of mRNA levels of IGF‐IR, total IR, and isoforms IRa and IRb in S‐ASC and V‐ASC. mRNA copy numbers/μL of cDNA were quantified by absolute qPCR using standard curves for IGF‐IR, IR‐tot, IRa, and IRb in S‐ASC and V‐ASC. (a, b) Quantification of copy numbers of IGF‐IR, total IR, IRa, and IRb in S‐ASC (*n* = 18) and in V‐ASC (*n* = 18) from human donors. (c, d) Comparison of transcript abundance of IGF‐IR, IR‐tot, IRa, and IRb between S‐ASC and V‐ASC. Data are shown as box and whisker plots. Boxes represent the interquartile range (IQR, 25th–75th percentiles); the horizontal line indicates the median; whiskers represent the minimum and maximum values; filled circles indicate individual observations. Statistical analysis for panels a and b was performed using Kruskal‐Wallis test followed by Dunn's multiple comparisons test. Statistical analysis for panels c and d was performed using two‐way ANOVA followed by Sidak's multiple comparisons test. *p < 0.05; **p < 0.01; ****p < 0.0001 for indicated comparisons.

Depot‐specific comparisons revealed significantly greater IGF‐IR and IR‐tot abundance in S‐ASC compared to V‐ASC (IGF‐IR: *p* < 0.01; IR‐tot: *p* < 0.05; Figure 1c). Similarly, IRa expression was significantly lower in V‐ASC relative to S‐ASC (*p* < 0.01), whereas IRb levels did not differ significantly between precursors (Figure 1d).

### 3.2 Activation of Insulin Signaling in S‐ASC and V‐ASC

To evaluate insulin responsiveness, phosphorylation of Akt at both Thr^308^ and Ser^473^ residues, as well as Erk phosphorylation at Thr^202^ and Tyr^204^ sites, was assessed following acute stimulation with increasing concentrations of human insulin (0–1000 nM, 15 min; Figure 2a–c, left panels; Figure 2d).

**FIGURE 2:**
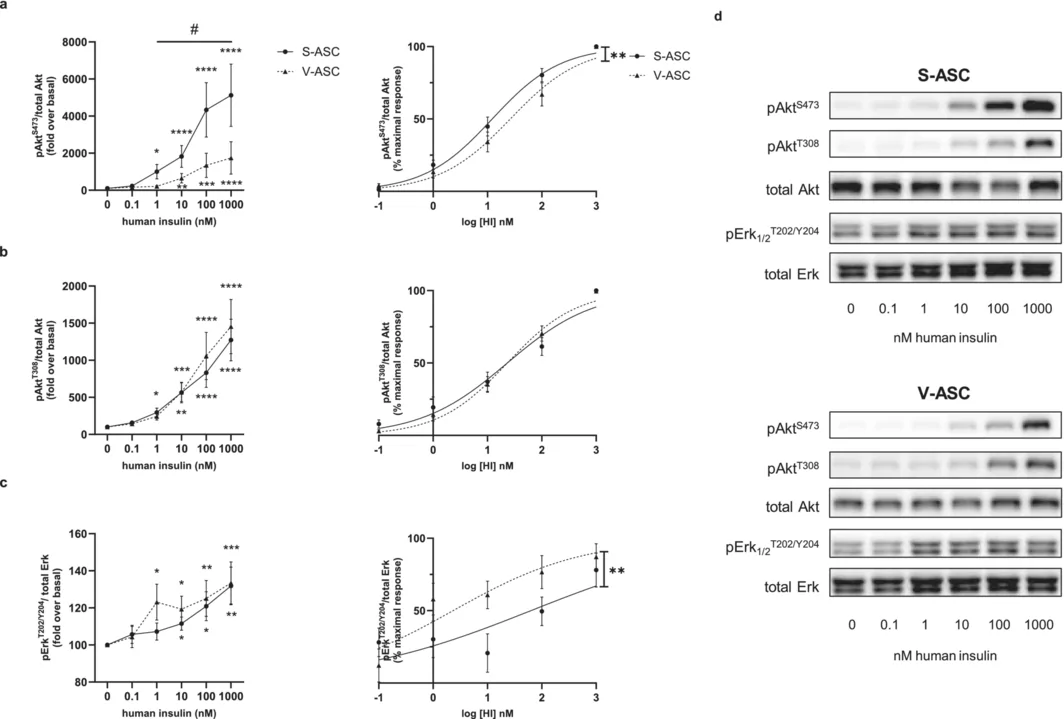
Insulin signaling in S‐ASC and V‐ASC. Dose–response curves for (a) pAkt^S473^/total Akt, (b) pAkt^T308^/total Akt, and (c) pErk^T202/Y204^/total Erk1/2 in S‐ASC and V‐ASC stimulated with increasing concentrations of human insulin (HI) (0–1000 nM, 15 min), expressed as fold over basal (left panels) and as percentage of maximal response (right panels). (d) Representative Western blot images for pAkt^S473^, pAkt^T308^, total Akt, pErk^T202/Y204^, and total Erk in S‐ASC and V‐ASC stimulated with increasing concentrations of human insulin (0, 0.1, 1, 100, and 1000 nM). Data are expressed as mean ± SEM. S‐ASC: *N* = 11; V‐ASC: *N* = 9. Filled circles, S‐ASC; open triangles, V‐ASC. Statistical analysis of dose‐dependent phosphorylation responses within each depot was performed using Kruskal–Wallis test followed by Dunn's multiple comparisons test. *p < 0.05; **p < 0.01; ***p < 0.001; ****p < 0.0001 versus basal (0 nM). Differences between S‐ASC and V‐ASC at each insulin concentration were assessed by Mann–Whitney test with Holm‐Sidak correction for multiple comparisons (^#^ *p* < 0.05 vs. V‐ASC at the same concentration of insulin). EC_50_ values were calculated by nonlinear regression using a four‐parameter logistic model with bottom = 0 and top = 100 constrained; comparison of EC_50_ between S‐ASC and V‐ASC was performed by *F*‐test (**p < 0.01).

A dose‐dependent increase in pAkt^S473^, pAkt^T308^, and pErk^T202/Y204^ was found in both S‐ASC and V‐ASC after insulin exposure. Notably, the three phosphorylation targets differed markedly in the magnitude of the response as well as in a depot‐specific manner.

Activation of the Akt pathway was pronounced at both phosphorylation sites. At Ser^473^, insulin significantly increased phosphorylation in both depots (S‐ASC: Kruskal‐Wallis test *H* = 45.57, *p* < 0.0001; V‐ASC: *H* = 32.59, *p* < 0.0001), with a significantly greater response in S‐ASC compared to V‐ASC at all concentrations ≥ 1 nM (*p* < 0.05; Figure 2a, left panel; Figure 2d), reaching median maximal responses of 2790‐fold (S‐ASC) versus 720‐fold (V‐ASC) at 1000nM insulin.

Dose–response analysis confirmed a significantly lower EC_50_ for pAkt^S473^ in S‐ASC compared to V‐ASC (12.22 nM, 95% CI: 8.6–17.2 vs. 26.25 nM, 95% CI: 16.1–42.2; *F*‐test *p* = 0.0097), indicating that subcutaneous‐derived cells reach half‐maximal Akt Ser^473^ activation at roughly half the insulin concentration required by visceral‐derived cells (Figure 2a, right panel).

At Thr^308^, insulin elicited a comparable dose‐dependent increase in both depots (S‐ASC: *H* = 47.39, *p* < 0.0001; V‐ASC: *H* = 39.42, *p* < 0.0001; Figure 2b, left panel; Figure 2d), with no significant differences between both progenitors at any dose, consistent with the absence of a significant difference in EC_50_ (23.37 nM, 95% CI: 13.10–41.21 vs. 23 nM, 95% CI: 15.87–33.12; *F*‐test *p* = 0.96; Figure 2b, right panel). Thus, the higher insulin sensitivity of S‐ASC was confined to the Ser^473^ site.

Intra‐depot comparison revealed that, within S‐ASC, Ser^473^ phosphorylation was significantly greater than Thr^308^ at concentrations ≥ 10 nM, with a significantly lower EC_50_ for pAkt^S473^ compared to pAkt^T308^ (12.22 nM, 95% CI: 8.63–17.28 vs. 23.37 nM, 95% CI: 13.10–41.21; *F*‐test *p* = 0.046; Figure S1c). Conversely, no significant differences between the two phosphorylation sites were detected in V‐ASC (pAkt^S473^: 26.25 nM, 95% CI: 16.12–42.24 vs. pAkt^T308^: 23.00 nM, 95% CI: 15.87–33.12; *F*‐test *p* = 0.897; Figure S1b,d), suggesting that the differential regulation of Ser^473^ and Thr^308^ phosphorylation is a feature specific to subcutaneous progenitors.

Erk activation followed a parallel but attenuated pattern. Insulin induced a modest yet significant dose‐dependent rise in pErk^T202/Y204^ in both depots (S‐ASC: *H* = 14.75, *p* = 0.01; V‐ASC: *H* = 23.01, *p* = 0.0003; Figure 2c, left panel; Figure 2d), with maximal responses (~133‐fold) an order of magnitude smaller than those of Akt, without significant differences between depots at any concentration. Notably, dose–response analysis revealed a significantly lower EC_50_ for pErk^T202/Y204^ in V‐ASC compared to S‐ASC (2.334 vs. 24.03 nM; *F*‐test *p* = 0.007; Figure 2c, right panel), indicating that visceral progenitors achieve half‐maximal Erk activation at substantially lower insulin doses despite comparable maximal responses.

To explore whether receptor abundance influenced insulin‐induced signaling, ASC were stratified into high‐ and low‐expression groups based on median IR and IGF‐IR mRNA levels.

In S‐ASC, higher transcript levels of IGF‐IR, IR‐tot, and IRa were associated with significantly greater Akt Ser^473^ activation (*p* < 0.01; Figure S2a), whereas no significant association was detected in V‐ASC for any transcript analyzed (Figure S2b), suggesting a depot‐specific relationship between receptor expression and insulin‐induced Akt Ser^473^ phosphorylation.

### 3.3 Quantification of IGF‐IR, IR‐tot, IRa, and IRb mRNA Transcript Levels During Adipogenic Differentiation

ASC from both depots were induced to differentiate into adipocytes (dASC). Differentiation efficiency was markedly greater in S‐ASC (~60%) than in V‐ASC (~20%) as assessed by Nile Red/DAPI staining (Figure S3a). Oil‐Red‐O staining confirmed greater lipid accumulation in S‐dASC compared with V‐dASC (Figure S3b).

Absolute transcript quantification revealed distinct depot‐specific patterns (Figure 3a,b). IGF‐IR expression significantly decreased in S‐dASC relative to S‐ASC (6.8 × 10^3^ ± 1.4 × 10^3^ vs. 1.5 × 10^4^ ± 0.20 × 10^4^ copies/μL; *p* < 0.0001) but remained unchanged in V‐ASC upon differentiation (~1.3 × 10^4^ ± 0.18 × 10^4^ vs. 1.0 × 10^4^ ± 0.13 × 10^4^ copies/μL in V‐dASC vs. V‐ASC), resulting in significantly higher IGF‐IR levels in V‐dASC compared to S‐dASC (*p* < 0.05; Figure 3a). Otherwise, IR‐tot copy numbers increased significantly upon differentiation in both depots (S‐dASC vs. S‐ASC: 1.0 × 10^4^ ± 0.14 × 10^4^ vs. 4.2 × 10^3^ ± 0.54 × 10^3^ copies/μL; *p* < 0.01; V‐dASC vs. V‐ASC: 7.4 × 10^3^ ± 0.8 × 10^3^ vs. 2.7 × 10^3^ ± 0.37 × 10^3^ copies/μL; *p* < 0.05), reaching higher levels in S‐dASC as compared to V‐dASC (*p* < 0.05; Figure 3a).

**FIGURE 3:**
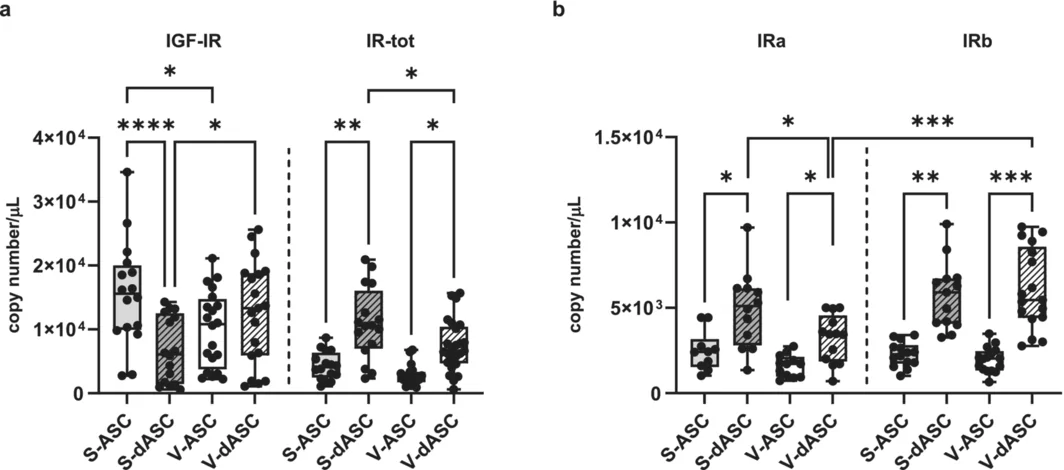
Quantification of mRNA levels of IGF‐IR, total IR, and isoforms IRa and IRb in ASC before and after adipogenic differentiation. (a) IGF‐IR and IR total copy numbers/μL were compared before (S‐ASC and V‐ASC) and after adipogenic differentiation (S‐dASC and V‐dASC) in subcutaneous and visceral depots. (b) IRa and IRb copy numbers/μL were compared before (S‐ASC and V‐ASC) and after adipogenic differentiation (S‐dASC and V‐dASC) in subcutaneous and visceral depots. Data are shown as box and whisker plots. Boxes represent the interquartile range (IQR, 25th–75th percentiles); the horizontal line indicates the median; whiskers represent the minimum and maximum values; filled circles indicate individual observations (S‐ASC and S‐dASC: *N* = 18; V‐ASC and V‐dASC: *N* = 18). Statistical analysis was performed using two‐way ANOVA followed by Sidak's multiple comparisons test. *p < 0.05; **p < 0.01; ***p < 0.001; ****p < 0.0001 for indicated comparisons.

IRa increased significantly in both depots, reaching higher levels in S‐dASC than in V‐dASC (4.7 × 10^3^ ± 0.47 × 10^3^ vs. 2.8 × 10^3^ ± 0.29 × 10^3^ copies/μL; *p* < 0.05; Figure 3b). IRb increased significantly in both depots after adipogenesis (*p* < 0.0001), with no significant difference between two differentiated precursors (S‐dASC: 5.8 × 10^3^ ± 0.63 × 10^3^ vs. V‐dASC: 6.0 × 10^3^ ± 0.57 × 10^3^ copies/μL; Figure 3b). Within depots, IRb exceeded IRa in V‐dASC (6.0 × 10^3^ ± 0.57 × 10^3^ vs. 2.8 × 10^3^ ± 0.29 × 10^3^ copies/μL; *p* < 0.0001), but not in S‐dASC (Figure 3b).

### 3.4 Quantification of IGF‐IR, IR‐tot, IRa, and IRb mRNA Transcripts in S‐AT and V‐AT

To assess whether the gene expression patterns observed in S‐ASC and V‐ASC were retained in intact AT, mRNA levels of IGF‐IR, IR‐tot, and IR isoforms were measured in paired S‐AT and V‐AT biopsies by absolute qPCR.

IR‐tot expression was significantly higher than IGF‐IR in both depots (S‐AT: 1.3 × 10^4^ ± 0.38 × 10^4^ vs. 2.0 × 10^3^ ± 0.71 × 10^3^ copies/μL; *p* < 0.05; V‐AT: 2.2 × 10^4^ ± 0.53 × 10^4^ vs. 4.2 × 10^3^ ± 1.2 × 10^3^ copies/μL; *p* < 0.01; Figure 4a). No significant differences were observed between S‐AT and V‐AT for either IGF‐IR or IR‐tot, or between IRa and IRb isoforms, although a trend toward higher IRb was observed in both depots (S‐AT: 2.5 × 10^3^ ± 0.81 × 10^3^ vs. IRa: 1.4 × 10^3^ ± 0.47 × 10^3^ copies/μL; V‐AT: 5.0 × 10^3^ ± 1.0 × 10^3^ vs. IRa: 2.6 × 10^3^ ± 0.65 × 10^3^ copies/μL; Figure 4b).

**FIGURE 4:**
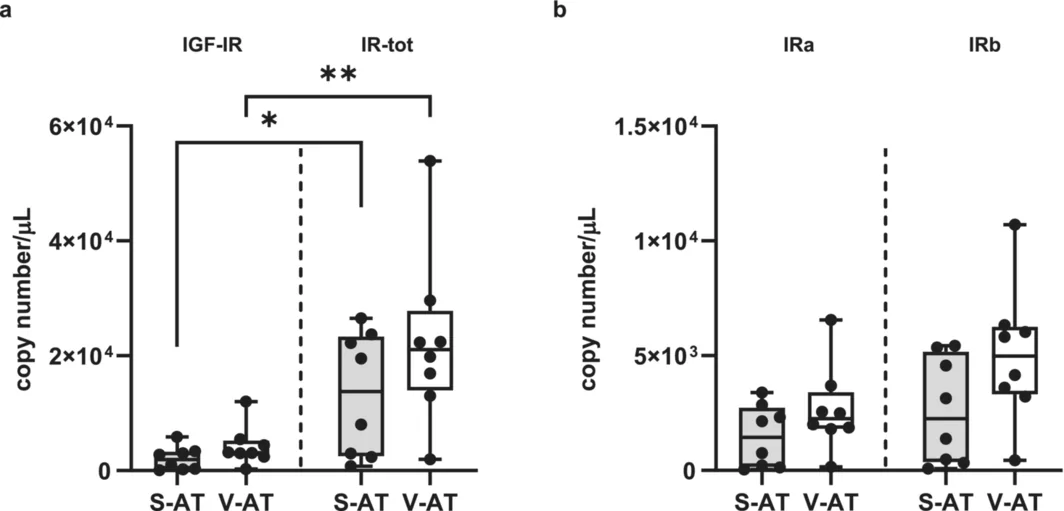
Quantification of mRNA levels of IGF‐IR, total IR, and isoforms IRa and IRb in human subcutaneous and visceral adipose tissue. Absolute transcripts copy numbers/μL were measured by standard‐curve qPCR in total RNA from paired subcutaneous (S‐AT) and visceral (V‐AT) adipose tissue samples from human donors (*n* = 8 per depot). (a) Comparison of IGF‐IR and IR‐tot transcript abundance between S‐AT and V‐AT. (b) Comparison of IRa and IRb transcript abundance between S‐AT and V‐AT. A dashed vertical line separates the two transcripts within each panel for clarity. Data are shown as box and whisker plots. Boxes represent the interquartile range (IQR, 25th–75th percentiles); the horizontal line indicates the median; whiskers represent the minimum and maximum values; filled circles indicate individual observations. Statistical analysis was performed using two‐way ANOVA followed by Sidak's multiple comparisons test. *p < 0.05; **p < 0.01 for indicated comparisons.

### 3.5 Analysis of IR Protein Expression During Adipogenesis of S‐ASC and V‐ASC

Flow cytometry with an anti‐IR antibody and Nile Red staining was used to evaluate IR protein expression during adipogenic differentiation.

In S‐dASC, the percentage of IR^+^ cells rose progressively from 35% ± 6.1% at Day 0 to 60% ± 8.6% at Day 30 (*p* < 0.05), with S‐dASC showing significantly higher IR^+^ cell proportions compared to V‐dASC at Day 30 (*p* < 0.01; Figure 5a). In V‐dASC, IR^+^ cells increased only transiently at Day 16 (~50% ± 4.8%) and returned to baseline levels by Day 30 (25% ± 5.5%).

**FIGURE 5:**
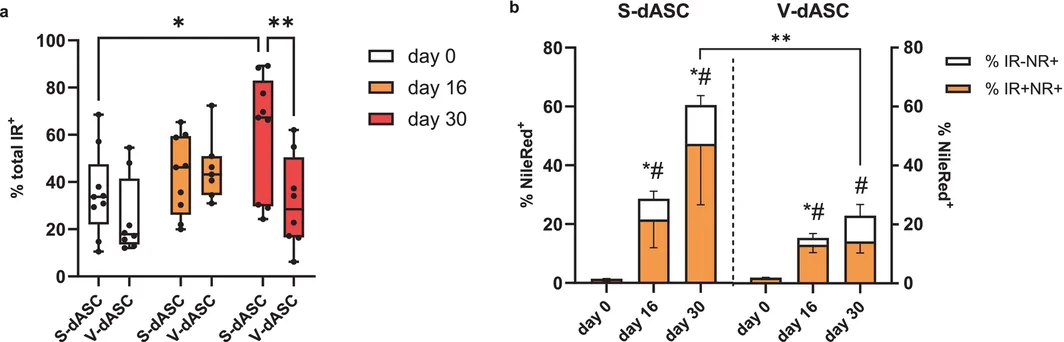
Cell‐surface IR protein during adipocyte differentiation. (a) Proportion of IR^+^ cells (% total IR^+^) in S‐dASC and V‐dASC at Day 0, 16, and 30 of adipogenic differentiation (S‐dASC: *N* = 9; V‐dASC: *N* = 8). Data are shown as box and whisker plots. Boxes represent the interquartile range (IQR, 25th–75th percentiles); the horizontal line indicates the median; whiskers represent the minimum and maximum values; filled circles indicate individual observations. Statistical analysis was performed using two‐way ANOVA followed by Sidak's multiple comparisons test. *p < 0.05; **p < 0.01 for indicated comparisons. (b) Proportion of IR^+^ Nile Red^+^ (% IR^+^NR^+^, filled orange bars) and IR^−^ Nile Red^+^ (% IR^−^NR^+^, white bars) cells in S‐dASC (left, *n* = 9) and V‐dASC (right, *n* = 8) at Day 0, 16, and 30 of adipogenic differentiation. S‐dASC: IR^+^NR^+^ cells represented 17.74% ± 3.8% at Day 16 and 47.3% ± 6.9% at Day 30; IR^−^NR^+^ cells represented 9.4% ± 3.2% at Day 16 and 13.2% ± 3.2% at Day 30. V‐dASC: IR^+^NR^+^ cells represented 13.1% ± 2.5% at Day 16 and 14.2% ± 3.7% at Day 30; IR^−^NR^+^ cells represented 2.4% ± 1.3% at Day 16 and 8.7% ± 3.6% at Day 30. The proportion of IR^+^NR^+^ cells was significantly higher in S‐dASC compared to V‐dASC at Day 30 (**p < 0.01, two‐way ANOVA with Sidak's post hoc test). Data are shown as bar chart (mean ± SEM). Statistical analysis was performed using two‐way ANOVA followed by Sidak's multiple comparisons test. *p < 0.05 IR^+^NR^+^ versus IR^−^NR^+^ at the same time point; ^#^ *p* < 0.05 versus Day 0. [Color figure can be viewed at wileyonlinelibrary.com]

Notably, not all Nile Red^+^ (NR^+^) adipocytes expressed IR on the cell surface: only ~77% of S‐dASC and ~61% of V‐dASC were IR^+^ at the end of adipogenesis.

In S‐dASC, IR^+^NR^+^ cells increased significantly from Day 0 to 16 (17.74% ± 3.8%) and Day 30 (47.3% ± 6.9%), while IR^−^NR^+^ cells remained consistently lower at both time points (9.4% ± 3.2% at Day 16; 13.2% ± 3.2% at Day 30; *p* < 0.05 IR^+^NR^+^ vs. IR^−^NR^+^; *p* < 0.05 vs. Day 0). In V‐dASC, IR^+^NR^+^ cells also increased over time (13.1% ± 2.5% at Day 16; 14.2% ± 3.7% at Day 30), but to a significantly lesser extent than in S‐dASC at Day 30 (*p* < 0.01 vs. S‐dASC; Figure 5b). A growing fraction of IR^−^NR^+^ cells was observed particularly in V‐dASC (2.4% ± 1.3% at Day 16; 8.7% ± 3.6% at Day 30; *p* < 0.05 vs. Day 0), indicating that a subset of adipocytes acquired lipid droplets regardless of detectable IR expression (Figure 5b).

## 4 Discussion

Insulin and its receptor are critical in the development of AT and play a significant role in metabolic regulation and its abnormalities such as type 2 diabetes. Despite extensive research on IR signaling, no studies have performed absolute quantification of IGF‐IR and IR isoforms in AT or during human adipogenesis. In this study, we report for the first time a thorough analysis of copy numbers of IGF‐IR, IR, and the IR splice variants in ASC and dASC of S‐AT and V‐AT, as well as in whole AT biopsies, from human donors by using absolute qPCR analysis.

Consistent with previous results [22, 23, 24], IGF‐IR was the most abundant transcript in both S‐ASC and V‐ASC as compared with IR‐tot, whose levels were significantly higher in S‐ASC than V‐ASC. The predominance of IGF‐IR over IR in undifferentiated progenitors is coherent with the established role of IGF‐IR in promoting proliferation and self‐renewal of mesenchymal stem cells [22, 23, 24]. After adipocyte commitment, IGF‐IR showed a significant downregulation in S‐dASC but not in V‐dASC, whereas IR‐tot was increased in both S‐dASC and V‐dASC with a greater amount at the end of subcutaneous adipogenesis. This mutual regulation of IGF‐IR and IR during adipogenesis is similar to the well‐documented receptor switch observed in 3T3‐L1 cells, in which preadipocytes respond predominantly to IGF‐I while adipocyte differentiation results in enhanced IR expression and acquisition of insulin responsiveness [25].

The IGF‐IR sequence is known to share ~80% homology with IR, activating similar intracellular pathways and regulating both mitogenic and metabolic responses [26]. However, IGF‐I signaling in AT seems to play a role only in controlling somatic growth rather than being crucial for adipocyte development and differentiation as demonstrated in a mouse model of conditional adipose‐specific Igf1r gene suppression, where AT development and differentiation occurred normally [27]. Accordingly, the selective reduction in IGF‐IR expression in S‐dASC, but not in V‐dASC, may thus reflect a more thorough reprogramming of the receptor mRNA profile during subcutaneous adipogenesis, while visceral progenitors appear to retain progenitor‐like characteristics even following adipogenic induction.

Regarding IR isoforms, a significantly elevated copy number of IRa was found in S‐ASC compared to V‐ASC, whereas IRb transcript levels were approximately twofold higher than IRa in V‐ASC. The higher IRa abundance in S‐ASC is consistent with the known role of this isoform in actively proliferating and undifferentiated cells [10, 28, 29]. Considering the known metabolic role of IRb in adult tissues [30], the relative predominance of IRb in V‐ASC may therefore represent an early molecular hallmark of the depot‐specific metabolic properties of V‐AT [1].

Depot‐related differences in receptor expression levels in undifferentiated ASC appear consistent with the observed differences in insulin responsiveness. It is well known how Akt activation represents a key insulin signaling node in adipocytes principally involved in favoring de novo lipogenesis and glucose transport [31]. S‐ASC displayed greater insulin sensitivity compared to V‐ASC, as evidenced by significantly higher pAkt^S473^ levels and a lower EC_50_. Notably, this depot‐specific difference was confined to the Ser^473^ site, as EC_50_ values for pAkt^T308^ were similar between the two precursors. Intra‐depot comparison further revealed that, within S‐ASC, Ser^473^ phosphorylation was significantly superior to Thr^308^ activation at concentrations ≥ 10 nM, with a significantly lower EC_50_ for pAkt^S473^ compared to pAkt^T308^, a difference not detected in V‐ASC.

As suggested by Balasuriya et al. [32], Ser^473^ and Thr^308^ regulate distinct downstream targets: Ser^473^ activation preferentially regulates metabolic targets implicated in glucose metabolism and adipogenesis, including FOXO transcription factors and GSK3, while PDK1‐mediated Thr^308^ phosphorylation primarily activates substrates involved in cell survival and protein synthesis. The preferential and depot‐specific increase in Akt Ser^473^ phosphorylation in S‐ASC, together with the well‐established greater adipogenic capacity, suggests that insulin‐induced Akt Ser^473^ signaling may be a key determinant of the enhanced adipogenic potential of subcutaneous progenitors, consistent with the established role of Akt in promoting adipocyte differentiation and lipid accumulation [31, 33].

On the other hand, in adipose depots, particularly in mature adipocytes, the glucose transport induced by insulin occurs in a depot‐specific manner, with higher rates of glucose uptake in V‐AT compared to S‐AT [1]. Accordingly, the activation of insulin signaling is faster and more transient in V‐AT compared to S‐AT [34], patterns that are replicated in V‐dASC [14]. These observations appear to be at least partially conflicting considering the greater pAkt^S473^ observed in S‐ASC in vitro. While profound phenotypic changes occur during adipogenic differentiation, the intricate paracrine and endocrine interactions existing in the whole AT microenvironment are not fully recapitulated in cell culture models. Yet the selective pAkt^S473^ advantage in S‐ASC may be preferentially linked to promotion of adipogenic commitment rather than acute metabolic responses such as glucose transport.

Regarding the MAPK/ERK pathway, although no significant differences in pErk^T202/Y204^ levels were observed between S‐ASC and V‐ASC, dose–response analysis revealed a significantly lower EC_50_ in V‐ASC compared to S‐ASC. Whether this depot‐specific difference in Erk sensitivity carries functional consequences for adipogenesis remains an open question, as this pathway has been shown to exert a dual role during adipocyte differentiation, being required for the early mitotic clonal expansion of stem cells while needing to be downregulated for adipocyte maturation [35].

Taken together, these depot‐specific differences in receptor dynamics and signaling are ultimately mirrored at the functional level: S‐ASC displayed a higher adipogenic potential compared to V‐ASC [7, 8, 14, 29, 36, 37]. Adipogenesis was associated with downregulation of IGF‐IR in S‐dASC, possibly reflecting a negative feedback driven by increased IGF‐I production during adipogenic induction [25, 38], while IR‐tot increased in both depots as developing adipocytes become receptive to insulin [39, 40]. The more pronounced upregulation of IR‐tot in S‐dASC is consistent with the greater adipogenic efficiency of subcutaneous progenitors.

Upon differentiation, IRa increased in both depots, with a significantly greater upregulation in S‐dASC. Whereas S‐dASC maintained comparable levels of IRa and IRb, V‐dASC showed a significant predominance of IRb over IRa, consistent with data from murine visceral preadipocytes [41], suggesting that visceral adipogenesis is associated with a preferential shift toward the metabolic isoform.

The adipogenic competence of both S‐ASC and V‐ASC according to IR protein expression was evaluated by flow cytometry analysis: a time‐dependent increase of IR protein expression was observed in S‐dASC but not in V‐dASC. Interestingly, not all differentiated adipocytes expressed IR on the cell membrane. The presence of IR^−^NR^+^ cells, particularly in visceral cultures, indicates that a fraction of cells underwent adipocyte conversion regardless of IR expression, consistent with recent evidence in mice showing that ASC dynamics and adipocyte formation are not always dependent on IR [42]. The higher proportion of IR^−^NR^+^ cells in V‐dASC may therefore reflect a greater reliance on IR‐independent adipogenic pathways in visceral progenitors.

In whole AT specimens, IR exceeded IGF‐IR in both depots, different from what was observed in resident ASC, reflecting the well‐documented increase in IR expression that accompanies adipocyte maturation. No significant differences in IR isoform copy numbers were observed between depots, although a trend toward higher IRb levels was documented in both S‐AT and V‐AT, consistent with the metabolic role of IRb in insulin‐sensitive mature adipocytes [30]. In a rodent model, IR isoform expression underwent changes under obesogenic and diabetic conditions in a tissue‐dependent manner [43], suggesting that the isoform balance observed here may differ from that in metabolically healthy individuals, a limitation that warrants consideration. Additionally, it should be noted that transcript levels measured in whole AT may be influenced by its heterogeneous cellular composition, which varies among individuals and differs from the isolated ASC populations analyzed in the present study.

Although this study highlights novel clues regarding IR isoform distribution in human ASC and AT, some limitations need to be acknowledged. First, we did not measure the protein levels of IRa and IRb, as primary antibodies against these isoforms are currently unavailable. Therefore, we could only speculate based on results from qPCR analysis, assuming they reflect protein expression in the absence of significant post‐translational modifications. Second, even though we reported data regarding changes in IR isoforms after adipocyte maturation, we could not investigate copy numbers of IR as well as IR surface expression in isolated mature adipocytes, since the yield of mature adipocytes isolated from a few grams of AT is very low. Third, the effect of IGF‐I/II stimulation on adipogenesis was not evaluated even though a potential effect is plausible, considering the affinity of IGF‐I/II to IR (IRa > IRb) [44, 45] and their known roles in adipogenic regulation [12]. Finally, the lack of paired subcutaneous and omental biopsies from the same donor in all participants limits direct intraindividual comparison between the two adipose depots.

## Author Contributions

V.A.G. and A.C. were responsible for recruiting and characterization of human participants, designed experiments, analyzed the data, and wrote the paper. V.A.G., R.D., C.L., and C.C. performed experiments and analyzed the data; C.L, N.M, G.B., A.N., S.P., and L.L. critically reviewed the paper. F.G. was responsible for recruiting and characterization of human participants, designed experiments, analyzed the data, wrote the paper, and provided final editing. F.G. is the guarantor of this work and, as such, had full access to all the data in the study and takes responsibility for the integrity of the data and the accuracy of the data analysis.

## Funding

This work was supported by “CARDIOMET” (Fondazione Cianciola) and by “Type 2 diabetes, Obesity, and Chronic Stress as predisposing Network for Tumorigenesis” (PNRR‐MAD‐2022‐12375797).

## Conflicts of Interest

S.P.: AstraZeneca, Eli Lilly, Novo Nordisk, Sanofi Aventis, Menarini (honoraria); Novo Nordisk, Eli Lilly (participation on advisory boards). A.N.: Astra Zeneca, Lilly Boehringher, Lilly, Novo Nordisk, Sanofi Aventis (speaker fees). L.L.: Abbott, AstraZeneca, Boeringher‐Ingelheim, Eli Lilly Italia, Medtronic, MOVI SpA, Novo Nordisk, Roche Diabetes Care, Sanofi Aventis, Terumo (participation on advisory boards); Abbott, AstraZeneca, Boehringer‐Ingelheim, Eli Lilly, Merck Sharp & Dohme, Medtronic, Menarini, MOVI SpA, Mundipharma, Novo Nordisk, Roche Diabetes Care, Sanofi Aventis, Terumo (speaker fees). F.G.: Eli Lilly, Roche Diabetes Care (grants); Eli Lilly, Novo Nordisk (consulting fees); AstraZeneca, Boehringer‐Ingelheim, Eli Lilly, Lifescan, Merck Sharp & Dohme, Medtronic, Novo Nordisk, Roche Diabetes Care, Sanofi Aventis (support for attending meetings or travels); AstraZeneca, Boehringer‐Ingelheim, Eli Lilly, Lifescan, Merck Sharp & Dohme, Medimmune, Medtronic, Novo Nordisk, Roche Diabetes Care, Sanofi Aventis (participation on advisory boards); EASD/EFSD (unpaid leadership). A.C.: Astra Zeneca, Lilly, Novo Nordisk, Sanofi Aventis (speaker fees); Novo Nordisk (participation on advisory board). The other authors declared no conflicts of interest.

## Supporting information


**Figure S1:** Comparison of pAkt^S473^ and pAkt^T308^ in response to insulin stimulation in S‐ASC and V‐ASC. (a, b) Dose–response curves for pAkt^S473^/total Akt and pAkt^T308^/total Akt in S‐ASC (a) and V‐ASC (b) stimulated with increasing concentrations of human insulin (HI) (0–1000 nM, 15 min), expressed as fold over basal. (c, d) Dose–response curves for pAkt^S473^/total Akt and pAkt^T308^/total Akt expressed as percentage of maximal response in S‐ASC (c) and V‐ASC (d), fitted by nonlinear regression using a four‐parameter logistic model (bottom = 0 and top = 100 constrained). EC_50_ values were compared between pAkt^S473^ and pAkt^T308^ by *F*‐test. Data are expressed as mean ± SEM. S‐ASC: *n* = 11; V‐ASC: *n* = 9. Filled circles, pAkt^S473^; filled squares, pAkt^T308^. Differences between pAkt^S473^ and pAkt^T308^ at each insulin concentration were assessed by multiple Mann–Whitney tests with Holm‐Sidak correction for multiple comparisons. *p < 0.05; **p < 0.01; ***p < 0.001; ****p < 0.0001 versus basal (0 nM) for each phosphorylation site; ^#^ *p* < 0.05 pAkt^S473^ versus pAkt^T308^ at the same insulin concentration. EC^50^ comparison by *F*‐test between pAkt^S473^ and pAkt^T308^ (*p < 0.05).

**Figure S2:** Insulin‐induced Akt activation in S‐ASC and V‐ASC according to mRNA levels of IR and IGF‐IR. (a) Levels of pAkt^S473^/total Akt (fold over basal) in S‐ASC stratified according to high versus low expression of IGF‐IR, IR‐tot, IRa, and IRb (from left to right). (b) Levels of pAkt^S473^/total Akt (fold over basal) in V‐ASC stratified according to high versus low expression of IGF‐IR, IR‐tot, IRa, and IRb (from left to right). Data are expressed as mean ± SEM; individual observations are shown as filled circles and squares. Statistical analysis was performed using Mann–Whitney test. **p < 0.01 high versus low expression.

**Figure S3:** Quantification of adipocytes and lipid accumulation in dASC from S‐AT and V‐AT. (a) Quantitation of adipocyte conversion and (b) amount of accumulated lipids in S‐dASC (*n* = 9) and V‐dASC (*n* = 6). Magnification of representative microscopic images was ×10. Data are shown as box and whisker plots. Boxes represent the interquartile range (IQR, 25th–75th percentiles); the horizontal line indicates the median; whiskers represent the minimum and maximum values; filled circles indicate individual observations. Statistical significance was determined by unpaired two‐tailed Student's *t*‐test. *p < 0.05 S‐dASC versus V‐dASC.

**Table S1:** Anthropometric and biochemical characteristics of all participants who provided adipose tissue biopsies. FPI, fasting plasma insulin; FPG, fasting plasma glucose; HbA1c, glycated hemoglobin; HOMA‐IR, homeostatic model assessment‐insulin resistance.

**Table S2:** Primer sequences used for RT‐PCR.

**Table S3:** Gene block standards sequences used for qPCR analysis.

## Data Availability

The data that support the findings of this study are available on request from the corresponding author. The data are not publicly available due to privacy or ethical restrictions.
