# Supplementary material for: Depot‐Specific Insulin Receptor Isoform Dynamics During Human Adipose‐Derived Stem Cell Adipogenesis
Source: Obesity (Silver Spring). 2026 Oct 1;34(11):2124–34. doi: 10.1002/oby.70315 (PMC13716193; doi:10.1002/oby.70315)

Supplementary Figure 2

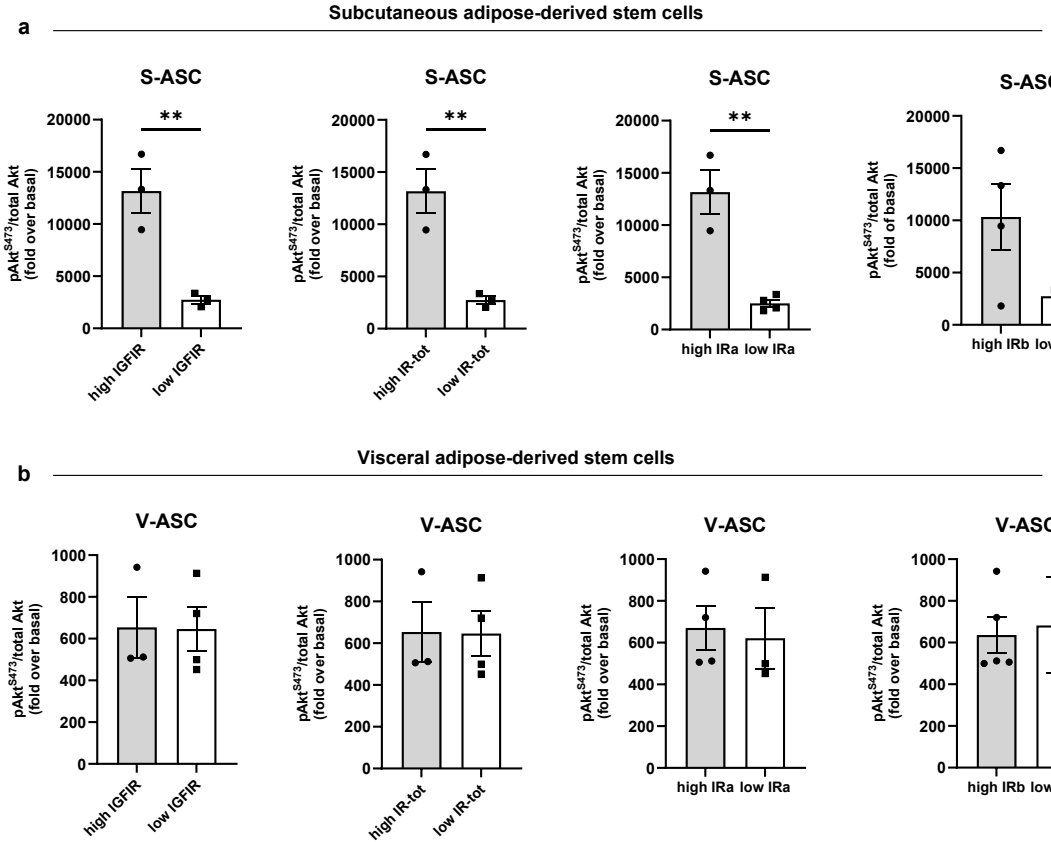

Supplement: Supplementary file 2 [file OBY-34-2124-s003.pdf]
