# Supplementary material for: Depot‐Specific Insulin Receptor Isoform Dynamics During Human Adipose‐Derived Stem Cell Adipogenesis
Source: Obesity (Silver Spring). 2026 Oct 1;34(11):2124–34. doi: 10.1002/oby.70315 (PMC13716193; doi:10.1002/oby.70315)
Supplement: Supplementary file 4 [file OBY-34-2124-s002.docx]

| Gene | Gene block sequence (5’-3’) | Concentration (copies/µL) |
| --- | --- | --- |
| IGF-IR | gaggagaagccgatgtgtgagaagaccaccatcaacaatgagtacaactaccgctgctggaccacaaaccgctgccagaaaatgtgcccaagcac | 7.34x10^11^ copies/µL |
| IR | caggagccgaaggagcccaatggtctgatcgtgctgtatgaagtgagttatcggcgatatggtgatgaggagctgcatctctgcgtctcccgcaagcac | 7.36x10^11^ copies/µL |
| IRa | gtggttttcgtccccaggccatctcggaaacgcaggtcccttggcgatgttgggaatgtgacggtggccgtgcccacggtggcagctttccccaacacttcctcgaccagcgtg | 7.95x10^10^ copies/µL |
| IRb | acgtggttttcgtccccagaaaaacctcttcaggcactggtgccgaggaccctaggccatctcggaaacgcaggtcccttggcgatgttgggaatgtgacggtggccgtgccca | 4.24x10^10^ copies/µL |

**Supplementary Table 3.** Gene block standards sequences used for qPCR analysis.
