# Supplementary material for: Depot‐Specific Insulin Receptor Isoform Dynamics During Human Adipose‐Derived Stem Cell Adipogenesis
Source: Obesity (Silver Spring). 2026 Oct 1;34(11):2124–34. doi: 10.1002/oby.70315 (PMC13716193; doi:10.1002/oby.70315)
Supplement: Supplementary file 5 [file OBY-34-2124-s005.docx]

| Gene | Accession No. | qPCR primers (5’-3’) | |
| --- | --- | --- | --- |
|  |  | Forward | Reverse |
| IGF-IR | NM_000875.4 | cgatgtgtgagaagaccacca | acattttctggcagcggttt |
| IR | NM_000208.4 | aggagcccaatggtctga | gagacgcagagatgcagc |
| IRa | NM_001079817.2 | tccccaggccatctcggaaa | gaggaagtgttggggaaagc |
| IRb | NM_000208.4 | cgtccccagaaaaacctcttc | caccgtcacattcccaacatc |

**Supplementary Table 2.** Primer sequences used for RT-PCR.
