# Supplementary material for: Depot‐Specific Insulin Receptor Isoform Dynamics During Human Adipose‐Derived Stem Cell Adipogenesis
Source: Obesity (Silver Spring). 2026 Oct 1;34(11):2124–34. doi: 10.1002/oby.70315 (PMC13716193; doi:10.1002/oby.70315)
Supplement: Supplementary file 6 [file OBY-34-2124-s006.docx]

| N | 54 |
| --- | --- |
| Age | 49 (±15.1) |
| Female | 36 |
| Male | 18 |
| BMI (kg/m^2^) | 35 (±7.9) |
| Waist (cm) | 109.6 (±18.5) |
| Weight (kg) | 94.1 (±20.9) |
| HOMA-IR | 5.9 (±5.6) |
| HbA1c (mmol/mol) | 54.8 (±31.7) |
| FPI (mIU/L) | 17.2 (±12.4) |
| FPG (mg/ml) | 113 (±39.2) |

**Supplementary Table 1.** Anthropometric and biochemical characteristics of all subjects provided adipose tissue biopsies. BMI, body mass index; HbA1c, glycated hemoglobin; HOMA-IR, homeostatic model assessment-insulin resistance; FPI, fasting plasma insulin; FPG, fasting plasma glucose.
